# Enhancement of the Physical and Mechanical Properties of Cellulose Nanofibril-Reinforced Lignocellulosic Foams for Packaging and Building Applications

**DOI:** 10.3390/nano14221837

**Published:** 2024-11-17

**Authors:** Mara Paulette Alonso, Rakibul Hossain, Maryam El Hajam, Mehdi Tajvidi

**Affiliations:** 1 Department of Chemical and Biomolecular Engineering, University of California, Berkeley, CA 94720, USA; mpalonso@ncsu.edu; 2 School of Forest Resources and Advanced Structures and Composites Center, University of Maine, Orono, ME 04469, USA; rakibul.hossain@maine.edu (R.H.); maryam.el1@maine.edu (M.E.H.)

**Keywords:** foam, insulation, packaging, cellulose, additive, building

## Abstract

Biobased foams have the potential to serve as eco-friendly alternatives to petroleum-based foams, provided they achieve comparable thermomechanical and physical properties. We propose a facile approach to fabricate eco-friendly cellulose nanofibril (CNF)-reinforced thermomechanical pulp (TMP) fiber-based foams via an oven-drying process with thermal conductivity as low as 0.036 W/(m·K) at a 34.4 kg/m^3^ density. Acrodur^®^, iron chloride (FeCl_3_), and cationic polyacrylamide (CPAM) were used to improve the foam properties. Acrodur^®^ did not have any significant effect on the foamability and density of the foams. Mechanical, thermal, cushioning, and water absorption properties of the foams were dependent on the density and interactions of the additives with the fibers. Due to their high density, foams with CPAM and FeCl_3_ at a 1% additive dosage had significantly higher compressive properties at the expense of slightly higher thermal conductivity. There was slight increase in compressive properties with the addition of Acrodur^®^. All additives improved the water stability of the foams, rendering them stable even after 24 h of water absorption.

## 1. Introduction

Anthropogenic carbon is the name given to greenhouse gas emissions—mostly composed of carbon dioxide (CO_2_)—that have been released since the inception of industrialization. In fact, human activity has caused CO_2_ emissions to increase by 40% in the last 170 years. The accumulation of CO_2_ in the atmosphere is one of the major reasons for human-induced climate change [1]. A primary contributor to carbon emissions is the combustion of fossil fuels like coal for energy production, underscoring the significance of decreasing energy consumption and transitioning to more environmentally friendly energy sources [2]. Globally, the building sector predominates energy consumption, accounting for approximately 37% of the total final energy consumption [3]. A large part of a building’s energy use goes toward managing heat exchange through its envelope, due to significant heat loss or gain through the walls, roofs, and ceilings. Therefore, any improvement in the thermal insulation of the construction is expected to lower energy consumption in buildings during use. Due to their light weight, cost-effective production, resistance to heat, water, and various chemicals, petroleum-based products have become increasingly popular in recent decades for use in thermal insulation applications [4]. Petroleum-based foams, such as expanded/extruded polystyrene and polyurethane, stand as the premier choice for thermal insulation materials in the construction industry due to their excellent thermal resistivity [5]. However, the environmental and health impacts of petroleum-based materials have emerged as a significant global concern [6]. The detrimental effects these substances have on ecosystems and human well-being underscore the urgent need for more sustainable alternatives.

Energy consumption in the packaging industry is at an all-time high, with the sector accounting for 40% of global plastic use, leading to substantial energy consumption [7]. Petroleum-based foams are extensively utilized in the packaging industry, particularly for their cushioning and protective properties. Cushioning packaging refers to a system designed to protect the enclosed product from damage during distribution and handling processes. There is a sharp rise in demand for cushioning packaging, driven by increased manufacturing and the growth of online shopping. Petroleum-based foams, including expanded polystyrene (EPS), polyurethane, polyethylene, and polypropylene, are favored for cushioning due to their light weight and excellent cushioning properties [8]. Synthetic foams derived from petroleum-based sources pose environmental risks due to their resource consumption and difficulties in their disposal [9]. Despite being theoretically recyclable, the recycling of materials like EPS packaging is low, hindered by the process’s high cost and time requirement and aggravated by their high volume and low bulk density [10]. Moreover, EPS’ widespread use in disposable packaging significantly contributes to plastic pollution, exacerbating the global issue of disposing of hundreds of millions of tons of plastics every year [11]. 

In building and packaging applications, the substitution of petroleum-based foams with greener alternatives, without compromising material performance, is a much-desired need of the decade. Lignocellulosic materials obtained from wood can be an effective replacement of petroleum-based materials to produce foams [12,13,14]. These foams are novel, eco-friendly, renewable, and porous materials of a low density with a broad range of engineering applications [15]. Cellulosic materials are biodegradable, and ‘the most abundant renewable compound’ in the biosphere [16]. Cellulose nanofibrils (CNFs), obtained from cellulose, are garnering considerable attention in the foam-forming technology, due to their dual ability of reinforcing long fibers and stabilizing the bubbles in the surfactant-assisted foam-forming process [12]. CNFs are composed of a network of nanofibers that are flexible and have a ranges of particle sizes in the nano and micron scales [16]. The high aspect ratio and high surface area are desirable properties of CNFs that allow for good adhesion and strong connections with lignocellulosic fibers [17]. 

Surfactants play a critical role in foam generation by reducing surface tension, which facilitates increased foam production. The effectiveness of a surfactant is influenced by its chemical structure, its ability to be adsorbed at the air–water interface, and its critical micelle concentration [18], a point beyond which micelles are formed and the surfactant becomes ineffective. In the surfactant-assisted foam-forming process, CNFs stabilize the foams by acting as a ‘pickering agent’. CNFs get positioned between bubbles, delay bubble coalescence, and prevent liquid from flowing out of the foam [19]. 

In the literature, there are reports on the fabrication of low-density, highly porous, and thermal resistive surfactant-assisted CNF-reinforced lignocellulosic foams via air-drying or oven-drying [12,15,18,20]. Some of the challenges associated with these foams are their low mechanical and cushioning property and poor stability in water. Though the mechanical properties could be increased by increasing the density, a compromise needs to be made with other properties such as thermal resistivity. These challenges can be overcome by tuning the cellular architecture via chemical and physical cross-linking, changing drying conditions, and by the incorporation of additives in the foam structure [21]. There is insufficient literature available on the successful fabrication of surfactant-assisted lignocellulosic foams via the oven-drying process that can fulfill the standard requirements of the mechanical properties of EPS insulation (Type XI) at a low density [22]. 

We hypothesize that it is possible to tune the thermomechanical and physical properties of the foams by incorporating different additives, which can improve the interactions between the lignocellulosic fibers in the foams without disrupting the three-dimensional foam structures. Acrodur^®^, a formaldehyde-free polyester binder made from polycarboxylic acid and polyalcohol, is valued for its versatility, easy processing, environmental friendliness due to non-corrosiveness, and ability to bond with cellulose’s hydroxyl groups in natural plant fibers through forming ester groups [23]. CPAM, a water-soluble polymer with an ultra-high molecular weight, is used to increase the mechanical strength of cellulosic materials through bridging effects, electrostatic interaction, and hydrogen bonding between its positively charged amino groups and cellulose’s hydroxyl groups [13]. Finally, it has been reported that various metal ions such as Fe^3+^ can improve the interactions between cellulose fibers by cross-linking them in metal ion-containing solutions [24]. The effect of these additives on the foamability, foam stability, physical, and thermomechanical properties of the surfactant-assisted lignocellulosic foams have not been investigated before. Ongoing research at the University of Maine’s Laboratory of Renewable Nanomaterials utilizes thermomechanical pulp (TMP) fibers as the long fiber, CNFs as the binding agent, and sodium dodecyl sulphate (SDS) surfactants as the foaming agent, but the effects of additives and drying methods have not yet been examined. The objectives of this research were to provide insight into the effect of the addition of different additives like Acrodur^®^, CPAM, and FeCl_3_ on the properties of the foams. A comprehensive study was conducted to improve the thermal insulation, water stability, and mechanical properties of the lignocellulosic foams for thermal insulation and packaging applications and to investigate the factors affecting their properties.

## 2. Materials and Methods

### 2.1. Materials

TMP fibers with 667 ± 705 µm length and 30 ± 14 µm width [25] at a moisture content of 10 ± 2%, were kindly supplied by TimberHP (TimberHP Inc., Madison, ME, USA). CNFs (made from bleached Kraft pulp, at 90% fines content and 3% solids content) were kindly supplied by the Process Development Center (PDC) at the University of Maine. The fibrillation process of producing CNFs is patented and documented elsewhere [26]. SDS, an anionic surfactant, was selected as the foaming agent in this research and was purchased from Sigma-Aldrich (St. Louis, MO, USA). Acrodur^®^ DS 3515 at 60 wt.% solids and CPAM (Percol^®^ 175) were purchased from BASF Corporation (Florham Park, NJ, USA). Anhydrous FeCl_3_ (98% crystalline) was purchased from Thermo Fisher Scientific (Waltham, MA, USA). 

### 2.2. Fabrication Process of the Lignocellulosic Foam

**Figure 1:**
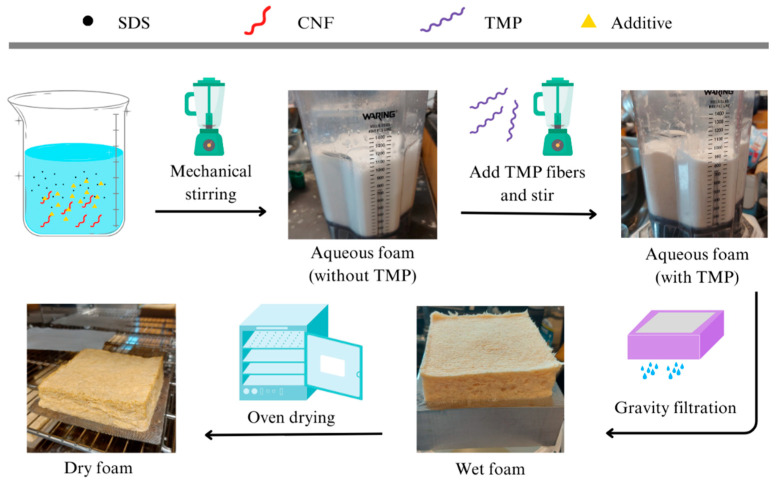
A schematic representation of the fabrication process of surfactant-assisted cellulose nanofibril-reinforced thermomechanical pulp fiber-based foams.

The foam-forming method used in this study was already optimized at the Laboratory of Renewable Nanomaterials at the University of Maine [25]. The selected formulation had a solids content (SC) of 7.5% and included fibers at a composition of 95 wt.% TMP and 5 wt.% CNFs as well as 2 g/L SDS were added to 200 mL of deionized water. Following the process depicted in Figure 1, the starting materials, including CNFs, water, SDS, and the respective additive, were foamed by mechanical stirring in the blender (Waring Commercial, Stamford, CT) for 3 min total, with 1 min at 2450 rpm and 2 min at 7400 rpm. The resulting aqueous foam product reached its maximum volume after stirring, showing a typical stable vortex. Consequently, TMP fibers were added to the blender and stirred for an additional 3 min at the same blender settings. The resulting aqueous foam was then transferred to a gravity filtration setup consisting of a square-shaped aluminum mold where it drained water for 10 min. The number of fibers lost during gravity filtration was negligible, and the pre-calculated dry mass of raw materials was almost equal to that of the dry foams. The wet-formed foam was finally placed in an air circulating oven and allowed to dry. Two different drying processes were tested on the neat (no additive) foams; the first one, called ‘low temperature drying’, included 12 h in the oven at 70 °C, while the ‘high temperature drying’ included an additional 6 h of drying at 105 °C. All the foams made with additives were dried by the ‘high temperature drying’ method. The dried foams were conditioned at a relative humidity of 50 ± 2% and temperature of 23 ± 2 °C for at least 24 h before performing any subsequent testing.

### 2.3. Characterization

To study the effect of additives on foam stability, aqueous foams with 2 g/L SDS content, 1% TMP, and 1% CNFs, as well as 1% of either Acrodur^®^, CPAM, or 1% FeCl_3_ in deionized water, were prepared using the same method as described in the previous section and added to a graduated cylinder. The amount of liquid collected at the bottom of the cylinder was measured for 10 min at an interval of 1 min to evaluate the effects of these constituents on foam stability. Aqueous foam stability was also tested for the formulations presented in Table 1 at 1% solids content. 

Foam volume (before and after TMP fiber addition) was observed from the graduated marks of the blender and the foamability was calculated according to Equation (1):

(1) $$Foamability\left(\%\right)=\frac{Volume\;of\;the\;aqueous\;foam}{Volume\;of\;the\;suspension\;before\;foaming}*100$$

The mass (m) of the preconditioned dry foams was measured in an analytical balance. The length (l), width (w), and thickness (t) of the foams were measured using a Vernier caliper and density (ρ) was calculated according to Equation (2).

(2) $$\rho =\frac{m}{l*w*t}$$

The density of 1500 kg/m^3^ was used for lignocellulosic fibers, and the porosity of the foams was calculated according to Equation (3).

(3) $$Porosity\left(\%\right)=\left(1-\frac{\rho }{1500}\right)*100$$

The surface and cross-sections of the foams were observed with a Zeiss Nvision 40 scanning electron microscope (Oberkochen, Germany). The foams were cryo-fractured in liquid nitrogen and the fractured samples were placed on specimen mounts with double-sided carbon tapes surrounded by copper tape. The samples were sputter coated with a 6 nm gold/palladium layer using a Cressington 108 auto sputter coater (Ted Pella Inc., Redding, CA, USA). The images were taken at an accelerating voltage of 20 kV, a distance range of 5.0–5.5 mm, and magnification of 2000×, 1000×, and 50× for each sample. 

To evaluate the distribution of the additives on the surface and cross-section of the foams, energy-dispersive X-ray spectroscopy (EDS) was performed using a Zeiss Nvision 40 SEM equipped with EDS (EDS, iXRF model 550i, Amray Inc., New Bedford, MA, USA). In this study, the distributions of carbon, nitrogen, sulfur, sodium, and iron were obtained, and the elemental mapping of nitrogen and iron were performed at 1000× magnification at both the surface and cross-section of the foams.

The water absorption (WA) and thickness swelling (TS) of the dry foams were measured according to the ASTM C1763 standard [27] with modified dimensions. The preconditioned mass (m_initial_) and thickness (t_initial_) of the foams were measured using an analytical balance. Tests were conducted for 2 h and 24 h of water absorption. The foams were taken out of the water and all the free water was left out to drain by gravity. Then, the foam was put in a few paper towels to remove the excess water before recording the mass (m_time_) and thickness (t_time_). Similarly, the same process was followed for 24 h. The WA and TS were calculated using Equations (4) and (5), respectively.

(4) $$WA\left(by\;volume,\%\right)=\left[\;\frac{\left(m_{time}-m_{initial}\right)}{m_{initial}}\;\cdot specific\;gravitiy\;of\;the\;foams\right]*100$$

(5) $$TS\;\left(\%\right)=\left[\;\frac{\left(t_{time}-t_{initial}\right)}{t_{initial}}\;\right]*100$$

The dry foams’ compressive properties were tested using an Instron (Model 5942, Instron Instruments, Norwood, MA, USA) equipped with a 500 N load cell and following the ASTM C165 [28]. The foams were cut into cylindrical shapes with a 50 mm diameter and varying thickness using a razor blade. The test was performed on conditioned samples at a speed of 0.89 mm/min. The compressive strength was reported at 10% and 25% compressive strain, but the test was continued up to 35% strain or until the maximum load reached 500 N (whichever came first). The thickness of the samples was measured within 1 min of the completion of the test at 35% strain and another reading was taken after 24 h. Thickness recovery for 1 min and 24 h was reported as the percentage of the final thickness to initial thickness. At least 8 replicates of each formulation were tested to determine the compression and thickness recovery properties.

To determine the interactions of the additives with the CNFs and the TMP fibers, films of the same formulations as Table 1 (without SDS) were prepared. The formulations were prepared at 1 wt.% SC and then filtered via a polystyrene mesh with a pore size of 70 microns using a vacuum filtration setup at 30 cm Hg pressure to filter the water. The wet mat was then taken out and placed between two filter papers and the entire assembly was sandwiched between two steel plates and pressed at 2.1 MPa pressure using a press (Carver Inc., Wabash, IN, USA) at room temperature. The pressed mat was then dried at 70 °C for 12 h in the oven, followed by an additional drying at 105 °C for 6 h. The dried circular films were cut into strips of 8 cm × 1.5 cm dimensions using a laser cutter and placed in the conditioning chamber for 24 h at a relative humidity of 50 ± 2% and temperature of 23 ± 2 °C. The conditioned strips were then tested using an Instron (Model 5942, Instron Instruments, Norwood, MA, USA). The Instron was equipped with an extensometer set at the gauge length of 10 mm and used to collect strain values during the tensile test. The crosshead speed and fixture separation were set to 2 mm/min and 40 mm, respectively, for the tensile tests. The fractured surfaces of the tensile specimens were then analyzed using an SEM.

Fourier transform infrared spectroscopy (FTIR) was conducted on the neat foams and foams with different additives (at 1% additive content) using a PerkinElmer FTIR-attenuated total reflectance (ATR) spectrometer (Spectrum Two, PerkinElmer, Shelton, CT, USA). The foam samples were placed on a diamond crystal equipped with an ATR accessory. Spectra were collected in absorbance mode with a resolution of 4 cm^−1^, using 16 scans over the 450–4000 cm^−1^. All spectra were normalized relative to the wavenumber 1055 cm^−1^, corresponding to the cellulose backbone’s stretching vibration.

The foam resilience of the preconditioned foams was measured using an IDM foam resilience tester (IDM Instruments Pty Ltd., Victoria, Australia). In this method, a 16 mm steel ball was dropped from 500 mm above the surface of the sample and the rebound height was measured. The resilience values range from 0 to 100% and the larger values mean better resilience. The ball drop test was recorded in slow motion and the exact percentage was obtained from the video. The test was performed at the four edges and at the middle of the foams and the average value of the percentage of the height dropped was reported. At least three replicates of each formulation were tested.

The thermal conductivity of the preconditioned foams was measured using an HFM 446 Lambda heat flow meter (NETZSCH-Gerätebau GmbH, Selb, Germany) according to ASTM C518 [29]. Each sample was tested at 2 temperature gradients, 10–30 °C and 20–40 °C, and the average of the 2 gradients was recorded. At least three replicates of each formulation were tested.

A one-way analysis of variance (ANOVA) was performed on the experimental data to determine significant differences between the foams made with different additives. The one-way ANOVA was followed by Duncan’s Multiple Range Test (DMRT) as a post hoc test. All statistical analyses were performed at a 95% confidence level using IBM SPSS Statistics Version 28 (IBM Corp., Armonk, NY, USA).

## 3. Results

### 3.1. Stability of Aqueous Foams by Drainage Test

**Figure 2:**
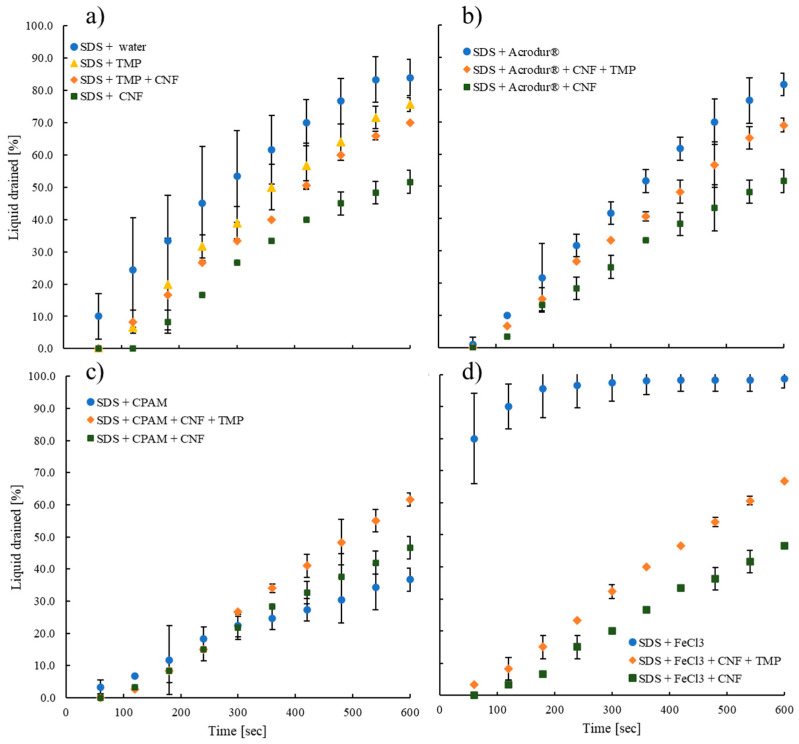
Stability of the aqueous foams (**a**) without any additive (neat foams), and with (**b**) 1% Acrodur^®^, (**c**) 1% CPAM, and (**d**) 1% FeCl_3_ as additives. All additives were added at 1% of total fibers (based on dry weight). When fibers were used, the composition was 95% TMP fibers and 5% CNFs.

The effects of the addition of the additives on the stability of the aqueous foams were investigated by the drainage test. Foam drainage is influenced by the downward liquid flow driven by gravity, the growth in bubble size from gas diffusion, and the rate at which foam breaks down due to bubble coalescence [30]. More water drainage means the foams are less stable. Comparing the drainage from aqueous foams (only containing water and SDS) in Figure 2a–d shows the effect of each additive on the foam stability. Figure 2a shows that the aqueous foam with no additive had poor stability, as water started draining in significant amounts at the 50 s mark. In terms of the aqueous foam in the presence of additives, Acrodur^®^ resulted in a slightly better foam stability than the foam without additives, draining less water at any given time. On the other hand, Figure 2d shows that foams with FeCl_3_ had the lowest stability at the 50 s mark, when almost 80% of the water had already been drained. This is possible as FeCl_3_ may reduce the surfactant content in foams by forming an insoluble complex, ferric dodecyl sulfate, which precipitates out, and destabilizes the foam [31]. Moreover, the Fe^3+^ ions can reduce the electrostatic repulsion between the SDS molecules, reducing their ability to produce a stable foam. On the contrary, Figure 2c shows that foams with CPAM as the additive were the most stable foams, where even at the 600 s mark, only 35% of the liquid had been drained. This may be attributed to the ability of hydrophilic CPAM to attach to the surface of the bubbles and prevent them from coalescing [32].

Next, an important observation in Figure 2a is that the aqueous foams containing water, SDS, and CNFs were the most stable foams in the absence of additives. This shows that CNFs stabilize the bubbles that are formed by the foaming agent SDS. The bubbles would normally fuse together, grow, and break, but in the presence of CNFs they form a Pickering emulsion-type structure that prevents neighboring bubbles from joining and breaking [20,33]. CNFs enhance the viscosity and elasticity of the air–water interphase around the bubbles and thus reduce the tendency of the bubbles to coalesce together [15]. Now, the effect of Acrodur^®^ on the aqueous foam (with CNFs only) increased the foam stability the most in Figure 2b, draining the least amount of water at any given time, because Acrudor^®^ contains carboxylic acid groups that interact with the hydroxyl groups present in CNFs forming hydrogen bonds [23] and may have a synergistic effect on stabilizing the bubbles. In the case of CPAM in Figure 2c, the aqueous foam containing CPAM only showed the highest stability compared to the ones with CNFs and CNFs+TMP. The addition of CNFs only increased stability during the first 300 s, after which the stability was very similar to the foams without CNFs. This is possible as CPAM contains carbonyl and amine groups, which may interact with the hydroxyl groups in CNFs via electrostatic interactions, causing flocculation of the fibers [34]. Consequently, comparatively fewer CNFs may be available at the surface of the bubbles, which may result in reduced stability of the foams (compared to CPAM + SDS only). On the other hand, for the aqueous foams containing FeCl_3_ as additives, the ones with CNFs had significantly higher stability compared to the ones without any fibers.

Finally, in all cases shown in Figure 2a–d, the addition of the TMP fibers resulted in a slight destabilization of the aqueous foam now containing both TMP and CNFs, compared to the foam containing CNFs only, due to the larger size and higher mass of the TMP fibers. Specifically, in Figure 2c, adding TMP fibers increased the water drainage, destabilizing the foam, because fibers flocculate in the presence of CPAM, allowing for more water drainage between the flocs [35]. However, for all additives, the aqueous foam containing both TMP and CNFs was still more stable than the aqueous foam with water only, showing that the addition of fibers, whether TMP or CNFs, increased the stability of the foams [30].

### 3.2. Foamability of the Aqueous Foams

**Figure 3:**
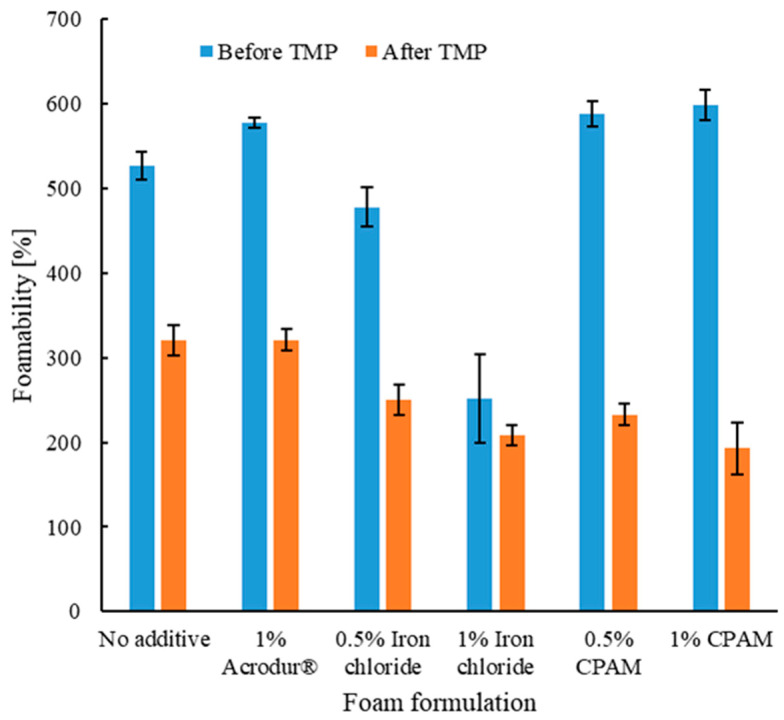
Foamability values before and after adding thermomechanical pulp (TMP) fibers during gravity filtration for different formulations for the preparation of the cellulose nanofibril-reinforced TMP fiber-based foams.

**Figure 4:**
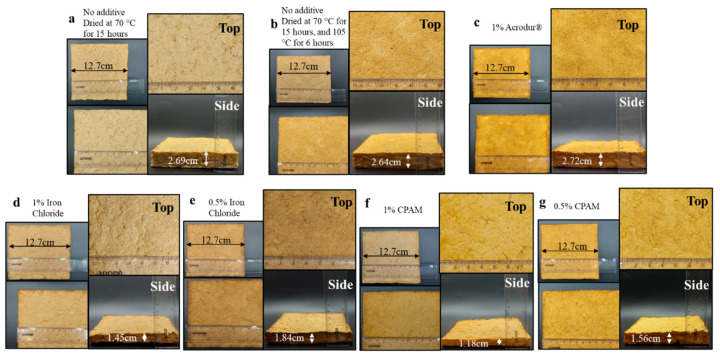
(**a**–**g**) Digital photographs of the cellulose nanofibril-reinforced thermomechanical pulp fiber-based foams of different formulations and their respective thicknesses.

Figure 3 shows the foamability of the mixture before and after adding TMP fibers. As expected, foamability values significantly decreased following the addition of TMP, given that TMP fibers constitute 95% of the fiber content at a consistency of 7.5% within the foams. The high consistency of the fibers in the foams leads to the inhibition and slowing down of the molecular movement of SDS molecules at the air–water interface [36]. This is an important step in the foam-making process as the reduced final volume of aqueous foam after the TMP fiber addition dictates the thickness of the dried foam. Figure 3 shows that the foamability before the TMP fiber addition was statistically the same in most formulations. However, in the case of FeCl_3_, the foaming was not as much as the rest of the formulations even before the addition of TMP fibers. This is possible as the addition of FeCl_3_ may reduce the overall surfactant content available for foam generation (as discussed in the previous section). FeCl_3_ was the only additive tested that also destabilized the foams and destroyed the bubbles that were trying to be formed due to the addition of SDS, a phenomenon also seen in the aqueous foam stability test presented in Figure 2d. Salts are known to increase the ionic strength of the solution, which can shield the electrostatic repulsion between the negatively charged CNFs, leading to aggregation, thereby reducing the efficiency of CNFs to contribute to bubble stability [37]. As for Figure 4, corresponding to the step during gravity filtration, the foams with 1% CPAM or FeCl_3_ had the least thickness. This is due to an increase in the destruction of bubbles in the presence of CPAM or FeCl_3_, thus decreasing the overall foam volume and resulting in a lower foam thickness. 

### 3.3. Morphology of the Dry Foams

**Figure 5:**
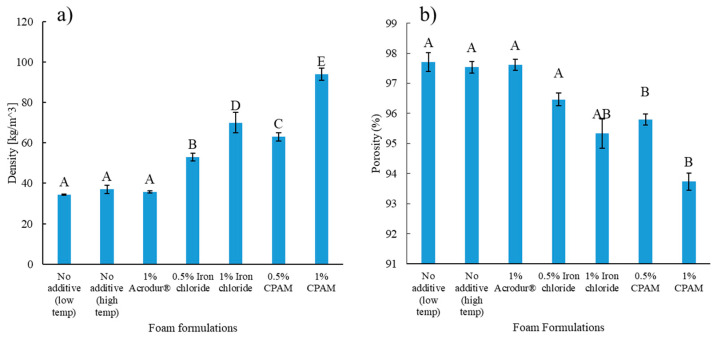
(**a**) Density and (**b**) porosity of the cellulose nanofibril-reinforced thermomechanical pulp fiber-based foams of different formulations. Values with common letters are not significantly different from each other at a significant level of 0.05.

In the digital photographs, the surface of the dry foams appeared to have a higher fiber network density than the inside (Figure 4). This implies a core–shell structure with a denser shell and softer core for these composite foams. A major takeaway is the difference in thickness across foams with different additives. Relating these results to Figure 4, it is observed that the foams showing the lowest thickness are those containing 1% FeCl_3_ and 1% CPAM, agreeing with previous results from Figure 3 showing the lowest foamability on aqueous foams with 1% FeCl_3_ and 1% CPAM. In Figure 4 and Figure 5a, we notice that the lower thickness foams are also the ones with the higher density, due to the decrease in volume. Therefore, the lower foamability during the foam-making process led to higher density on the dry foams. One important observation was that the foams did not experience a reduction in thickness after drying. Foams in Figure 4a–c had the highest thickness, showing that 1% Acrodur^®^ was the only additive that did not change the density significantly. This can be attributed to the foaming volume, as it was seen in Figure 3 that the addition of 1% Acrodur^®^ did not destroy the bubbles as the other additives did; hence, the foaming volume remained the same as the neat foams. This may be because Acrudor^®^ itself is a stable emulsion in water; therefore, the chemistry of the foam may not have changed after its addition. Figure 5a shows that foams with no additive (low and high temperature) and with 1% Acrodur^®^ had a density within 20–50 kg/m^3^, meaning that they fall under the ultra-low-density category of foams [38]. All foams had very high porosity and the values were within 94–98% (Figure 5b).

### 3.4. SEM, EDS, and FTIR Analysis

**Figure 6:**
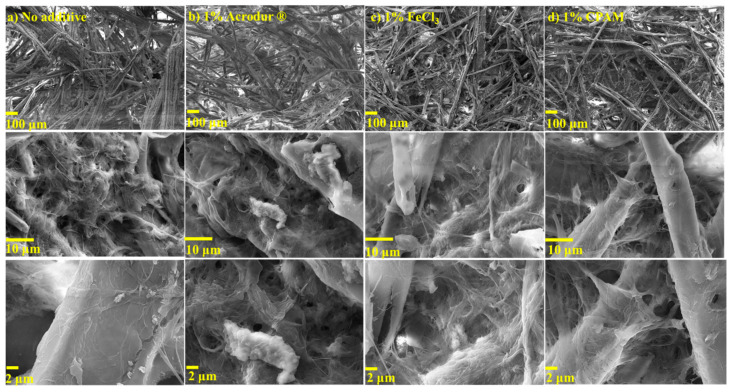
Scanning electron microscopy (SEM) images of the surfaces of the cellulose nanofibril-reinforced thermomechanical pulp fiber-based foams with (**a**) no additives, (**b**) 1% Acrodur^®^, (**c**) 1% FeCl_3_, and (**d**) 1% CPAM as additives.

SEM analysis was performed to investigate the internal structure of the fiber network and pores of the dry foams. SEM analysis revealed a random fiber orientation in all planes and a large proportion of pores in the fiber network for all formulations (Figure 6). In the traditional water-forming process, fibers naturally tend to align within the horizontal plane (xy-plane). In contrast, during the foam-forming process, fibers become “locked” onto the bubble surfaces. The spatial constraints imposed by the bubbles lead to a random orientation of the fibers throughout the foam structure [30]. The lowest magnification SEM images allowed for the observation of the large-sized TMP fibers, which are the backbone of the foam. SEM analysis revealed interconnected pores with open-cell pore structures in the foams. In Figure 6, the thinner and hair-looking CNFs can be seen as filamentous connections between large-sized TMP fibers. Due to CNFs being much smaller and branched, they allow for more interaction between the fibers through hydrogen bonding and mechanical interlocking [12]. Higher magnification images show film-like structures on the TMP fibers that may be formed due to the agglomeration of CNFs. Foams with additives Acrodur^®^, FeCl_3_, and CPAM at all additive dosages showed no considerable difference in the SEM images compared to neat foams (Figure 6).

The cross-sections of the foams showed a very porous structure to the naked eye. Cutting the foams with a blade was more challenging for the foams with a lower density. After cutting through the surface, the cross-section had fibers that fell apart from the rest of the foam structure. Nonetheless, as shown in Figure S1, there was no significant difference in SEM foam morphology at the cross-sections to that observed at the surface of the foam. 

**Figure 7:**
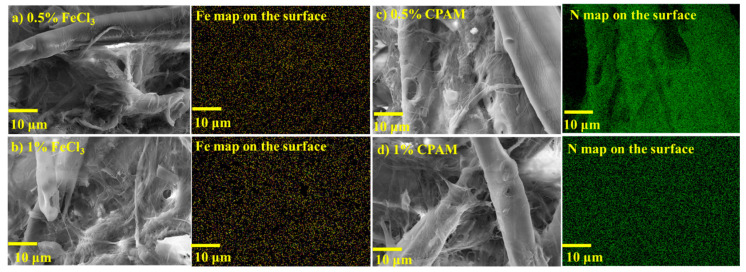
Energy-dispersive X-ray spectroscopy (EDS) images of the surface of the cellulose nanofibril-reinforced thermomechanical pulp fiber-based foams with (**a**) 0.5% FeCl_3_ (**b**) 1% FeCl_3_, (**c**) 0.5% CPAM, and (**d**) 1% CPAM as additives.

EDS images were obtained to observe the distribution of the main element in each of the additives in the foams. Figure 7 shows the EDS images on the surface of the foams. For foams with CPAM, nitrogen was the distinguishing element due to the presence of an amide functional group [39]. There was evidence of nitrogen along the surface of the samples (Figure 7). In the same way, FeCl_3_ as an additive showed the presence of iron on the surface. Similarly, reducing the amount of additive also reduced the presence of the element in the samples. EDS imaging of the foams’ cross-section showed nitrogen and iron across the cross-section (Figure S2). EDS analysis was not performed on the foams with Acrodur^®^, as the authors could not find any distinguishable element compared with those in the neat foams. 

The FTIR spectra of the neat foam and foams with different additives (at 1% additive content) are shown in Figure S3. The neat foam showed absorption peaks at 3330, 2920, 2848, and 1024 cm^−1^, which corresponds to the –OH groups, –CH stretching vibrations, and C–O–C stretching of the pyranose rings of cellulose, respectively [40]. The FTIR spectra of the foams with additives showed no significant differences compared to the neat foams, suggesting that no new chemical bonds were formed after adding a small amount of additives (1 wt.%). This indicates that the interactions between the additives and the fibers in the foams are likely to be predominantly physical.

### 3.5. Mechanical and Foam Resilience Properties

**Figure 8:**
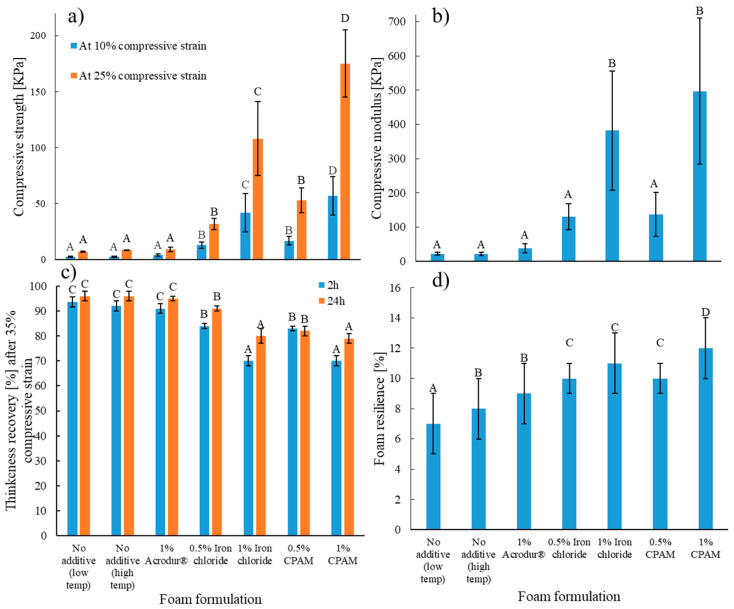
(**a**) Compressive strength at 10% and 25% strain, (**b**) compressive modulus, (**c**) thickness recovery after 35% compressive strain, and (**d**) foam resilience properties for cellulose nanofibrils-reinforced thermomechanical pulp fiber-based foams of different formulations. Values with common letters are not significantly different from each other at a significant level of 0.05.

The produced lignocellulosic dry foams should have sufficient mechanical strength and cushioning properties to be used for building and packaging applications. The mechanical and cushioning properties of the foams were evaluated by a compression test and foam resilience test, respectively. The compressive strength and modulus of the low-density lignocellulosic foams are strongly dependent on the density of the material, bending stiffness of the fibers, and the bonding between the fibers [30]. There was a power relationship of the compressive modulus with the density of the foams, where 67% of the variations in the compressive modulus could be explained by the variations in density (Figure S4). Foams with 1% FeCl_3_ and 1% CPAM performed better and had the highest compressive modulus and compressive strength at a 10% and 25% compressive strain compared to other formulations (Figure 8a,b). This can be attributed to the significantly higher density of these two groups of foams compared to other formulations. Such high compressive properties of these high-density foams may also be attributed to the decreased mean free fiber length of the fibers resulting from the increased number of joints enabled by the CNFs and the additives [30,41]. Neat dry foams had the lowest compressive properties, as they had the lowest density and the absence of additives, which meant there was no additional interactions among TMP fibers other than the bonding with CNFs. It was difficult to assess the difference across samples due to the significantly higher density in some formulations, which led to higher compressive properties. To compare foams with similar densities, a statistical analysis was performed on the samples with no additive (made at low and high temperature) and on the sample with 1% Acrodur^®^, where the latter showed a slight increase in the compressive properties, indicating that the additive did enhance the properties of the foam regardless of the density effect. This is possible as the carboxylic acid group of Acrodur^®^ and the hydroxyl groups in the fibers may form additional bonding in the foams [23]. There was no effect of the drying temperature on the compressive properties of the foams. To account for the effect of density, the specific modulus values of the foams were calculated. The results showed that, except for 1% Acrodur^®^, all additives enhanced the specific modulus to varying degrees (Figure S5), indicating a reinforcing effect of these additives on the foams. Overall, the foams with 1% CPAM and 1% FeCl_3_ could fulfill the minimum compressive strength requirement (35 kPa) at 10% strain for Type XI EPS insulation according to the standards [22]. 

In terms of thickness recovery, the foams in the lower range of density had up to 98% recovery and the values were significantly higher than the foams at higher density ranges (Figure 8c). This is possible as the lower density foams had much higher porosity in the fiber network and the fibers can bend with comparatively less geometric restrictions [42]. As a result, the fibers can avoid stress buildup that may lead to plastic deformation; thus, these fibers have a much higher thickness recovery. On the other hand, in the higher density foams, the fibers are more compactly arranged and may face more geometric restrictions when the compressive load is applied. Consequently, much more load is experienced by a relatively small number of fibers, which may cause plastic deformation and less thickness recovery [42].

The drop-ball rebound resilience test is commonly used to assess foam resilience following the ASTM D3574 standard [43]. A high foam resilience is especially important for cushioning packaging applications where the foam should keep its shape to protect a product from damage. Figure 8d shows the foam resilience values of the foams of different formulations. The average percentage of foam resilience recorded across samples in this study ranged from 7% to 11%. Previous research including the standardized ball-drop test on foams with a lignocellulosic component is limited. According to the literature, even commonly used foams like thermoplastic polyurethane are enhanced through microcellular foaming to increase mechanical properties such as foam resilience, leading to an average foam resilience of 60% [44]. Understandably, dry lignocellulosic foams need improvement in foam resilience, which is visibly shown in Figure 8d, where the foams with no additive are the least resilient to the ball-drop test and even were destroyed from the impact of the steel ball. The foams with 1% Acrodur^®^ also experienced damage at impact with the steel ball. The rest of the foams with additives did not show signs of damage after the test on their surface, indicating that the fibers were strong enough to avoid fracture. Evidently, the foams with the higher density also showed statistically higher foam resilience properties.

### 3.6. Tensile Properties of the Films

**Figure 9:**
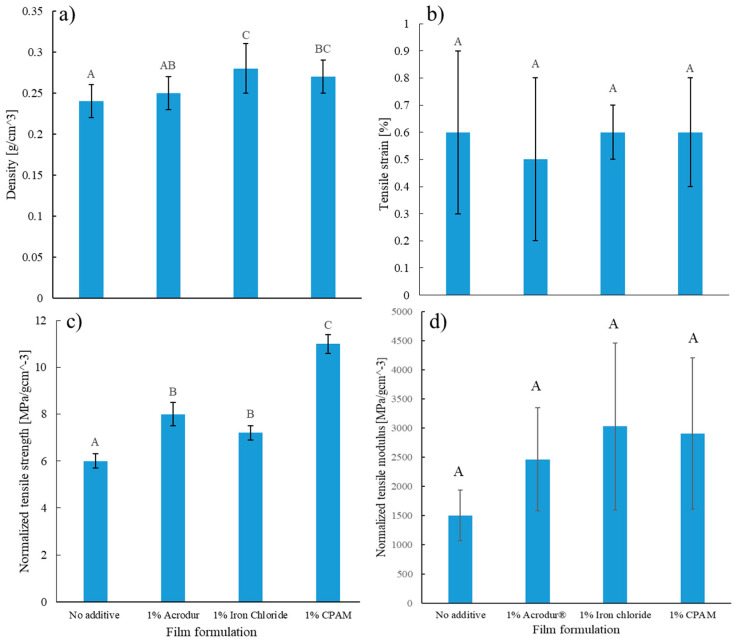
(**a**) Density, (**b**) tensile strain, normalized (**c**) tensile strength, and (**d**) tensile modulus of films (without SDS) with different additives. Values with common letters are not significantly different from each other at a significant level of 0.05.

To investigate the effect of the additives, without density interfering in the results of the mechanical properties of the lignocellulosic foams, films or fiber sheets were prepared using the same formulation as the foams but without the foaming agent SDS. A tensile test was performed to determine the mechanical properties of the films. Figure 9a shows that the films with additives had higher densities compared to the neat films. To eliminate the effect of density, the tensile strength and modulus of the films were normalized by dividing them by their respective densities. In Figure 9c, we observe three groups with a statistically significant difference, showing that the films with 1% CPAM had about a 46% higher tensile strength compared to the films without any additives. This is possible as CPAM may improve the fiber–fiber bond strength by promoting fiber–fiber interactions through electrostatic interactions between fibers and CPAM [13]. In addition to reinforcing long TMP fibers, the highly charged CPAM may also bring the negatively charged CNFs closer together, forming more joint points and further strengthening the network [45]. Films with 1% Acrodur^®^ and 1% FeCl_3_ also had statistically higher strength values than neat films but had lower values than the ones with 1% CPAM. As for 1% Acrodur^®^ and 1% FeCl_3_, they were not significantly different in their tensile strength. The carboxylic acid groups present in Acrodur^®^ may interact with the hydroxyl groups in cellulose, forming hydrogen bonds and covalent bonds for added strength [23]. Acrodur^®^ is also expected to facilitate the network formation of CNFs by interacting with their hydroxyl groups to potentially improve the mechanical strength of the films [46]. The addition of FeCl_3_ to the foams may improve their mechanical properties by the electrostatic interaction between the Fe^3+^ ion and the TMP fibers. Additionally, there is a possibility of the formation of an ionic cross-linking between the CNFs due to electrostatic attraction between the Fe^3+^ ion and carboxylate groups in CNFs [24]. Tensile strain and normalized tensile modulus values were not significantly different across films of different formulations (Figure 9b,d).

**Figure 10:**
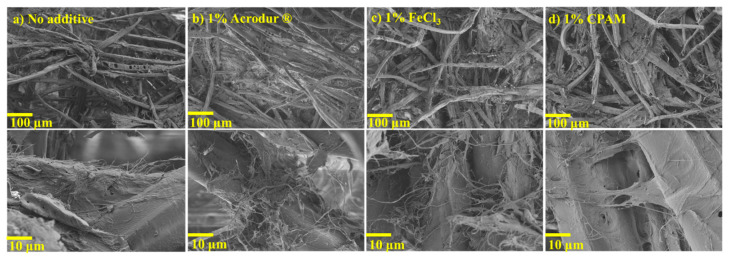
Scanning electron microscopy (SEM) images of the fractured surfaces of the cellulose nanofibrils-reinforced thermomechanical pulp fiber-based films with (**a**) no additive, (**b**) 1% Acrodur^®^, (**c**) 1% FeCl_3_, and (**d**) 1% CPAM as additives after tensile failure.

After tensile testing, the fractured surfaces of the composite films were prepared for SEM imaging. On the fractured surfaces of the films with additives, the fibers look ‘pulled apart’, which happened during failure (Figure 10). Comparatively more nanofibers were seen attached to the fibers with additives compared to the neat films. This may indicate that the additives improved the interaction between the fibers and CNFs. This would agree with findings in Figure 9c that the films with an additive had a significantly higher tensile strength than neat films. Furthermore, a second observation can be made regarding the voids observed. The film with no additive had voids in between the fibers, something that was not observed to the same extent on the other micrographs (Figure 10). The crosslinker Acrodur^®^ has been observed to fill the voids between the fibers in other studies (although the authors used 10% Acrodur^®^ as binder), which is also apparent here [47]. Foams with FeCl_3_ and CPAM also showed voids to a lesser extent, an indication of good adhesion between fibers which may lead to improved mechanical properties. Confirming the findings, other studies show that a larger pore size (also called voids) and fiber separation indicate low film integrity, which explains the low mechanical properties of the film with no additive [23]. From the tensile test and SEM analysis of the fractured surfaces of the films with and without additives, it is understood that the additives clearly played a role in reinforcing the films by promoting fiber–fiber bonding. It is an indication that in the case of the foams of the same formulations (with SDS), it is not only the density which is improving the mechanical properties but also the effect of the reinforcement by the additives. For a more comprehensive understanding of the role of additives in reinforcing the films, an in-depth investigation into the nanomechanical properties is essential [48]. Nevertheless, such an analysis falls beyond the scope of the present study.

### 3.7. Water Absorption and Thickness Swelling

**Figure 11:**
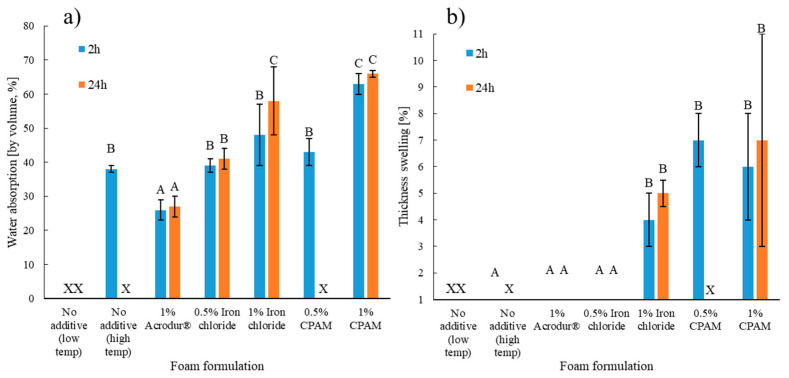
(**a**) Water absorption (by volume) and (**b**) thickness swelling of the cellulose nanofibril-reinforced thermomechanical pulp fiber-based foams of different formulations for 2 h and 24 h test times. ‘X’ indicates disintegration of foams after water absorption. Values with common letters are not significantly different from each other at a significant level of 0.05.

Water absorption is another important property in packaging and building applications. The mechanism of foam wetting starts with water diffusing into the pores, followed by the subsequent swelling and expansion of cellulose fibers [49]. CNFs form hydrogen bonds with TMP fibers during drying; when exposed to water, the fibrils swell up and the bonds between CNFs and TMP fiber are gradually broken down and replaced with hydrogen bonds between water and the hydroxyl groups of the fibers [50]. This explains the disintegration of the foams with no additives within 2 h of water absorption (Figure 11). The neat foams dried at a higher temperature; however, the neat foams experienced better water stability because they disintegrated after 24 h of water absorption. The better stability on the high temperature neat foams comes from the CNFs’ tendency to form a stronger bond with TMP fibers at higher temperatures, which may be due to ‘hornification’ [51]. Studies had previously suggested that networks of cellulose chain bonds were enhanced by rising temperatures [52]. To investigate this phenomenon, CNF suspensions were first dried at both low (70 °C for 6 h) and high temperatures (70 °C for 12 h + 105 °C for 6 h) and then left stirring in deionized water to test their water stability. The results confirmed that CNF films dried using the high temperature drying method took more than twice the time to break than those made using the low temperature drying method (Figure S6). 

It may be inferred that additives helped in the bonding of the TMP fibers, as TMP foams containing them did not disintegrate even after 24 h, except for foams containing 0.5% CPAM. The high density associated with the foams with additives allowed for more water absorption. One important observation was that for low-density foams, water could be easily removed from the foams by slight tilting. It is possible that the lower density foams had larger pores than the high-density foams and water could easily come out of them due to low capillary forces, resulting in lower water absorption values. As for the thickness swelling in Figure 11b, many foams did not show any thickness swelling, especially the lower density foams that did not disintegrate, most likely due to the foam being at its maximum expanded thickness. Since denser foams such as those with 1% FeCl_3_ and CPAM tend to bond the same number of fibers in a more compacted space, thickness swelling is expected during the water absorption. One important point to mention here is that though the addition of additives improved the water stability of the foams, the water absorption properties were still above the maximum limit (4% by volume) for the Type XI EPS insulation [22]. Further research needs to be conducted to improve the hydrophobic properties of the foams.

### 3.8. Thermal Conductivity of the Foams

**Figure 12:**
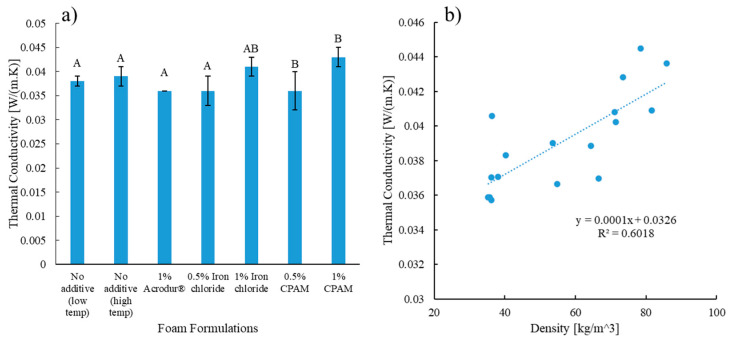
(**a**) Thermal conductivity and (**b**) the relationship between thermal conductivity and density of the cellulose nanofibrils-reinforced thermomechanical pulp fiber-based foams of different formulations. Values with common letters are not significantly different from each other at a significant level of 0.05.

Thermally insulating materials such as foams are a necessity for building applications, and improving the thermal insulation capacity is beneficial for energy saving. Thus, highly thermally insulating, and low-density lignocellulosic foams are a pathway to more sustainable buildings. TMP fibers have shown to be the best type of fibers for low thermal conductivity applications, as the results are better compared to hardwood fibers and softwood kraft pulp [30]. The porous structure of the foam affects the mechanism of heat transfer through the fibers. The closed cell structure of the hollow TMP fibers hinder convective heat transfer [18]. The restriction of the airflow from the TMP fibers lower the thermal conductivity, resulting in better thermal insulation. Another important aspect of heat transfer involves transfer through gas conduction due to the fraction of gas in the foams coming from the voids in it [41]. CNFs-based foams in past studies showed a similar trend that increasing density leads to a slight increase in thermal conductivity [41]. Similar phenomena were seen in this study, where thermal conductivity increased with the increase in the density of the foams, and the values ranged from 0.035 to 0.045 W/(m·K) (Figure 12a). There was a positive linear relationship between thermal conductivity and density, as 60% of the variations in thermal conductivity values could be explained by an increase in density (Figure 12b). This is possible as heat transfer via solid conduction increases due to increased solid fractions in the high-density foams [14].

Table 2 shows a comparison of the density and thermal conductivity values from various studies on both lignocellulosic foams and petroleum-based polymer foams. From Table 2, it is evident that at about the same density level, the produced foams in this study had comparable or lower thermal conductivity values compared to other biobased foams. Foams of all the formulations met the requirements of the maximum permissible thermal conductivity values for Type XI rigid cellular polystyrene thermal insulation products, as per the ASTM C578 [22].

## 4. Conclusions

CNF-reinforced, surfactant-assisted TMP fiber-based foams with various additives, developed through a simple oven-drying process, demonstrated significant improvements in performance. While the neat dry foams exhibited excellent thermal resistivity, they suffered from low mechanical strength, poor water stability, and limited resilience. The incorporation of different additives notably improved these properties to varying extents. All additives, except Acrodur^®^ at a 1% loading level, increased foam density, enhancing mechanical strength and water stability. Compression testing, FTIR analysis, and tensile tests on films of the same formulations (excluding SDS) provided evidence of fiber reinforcement through physical interactions within the foams. Despite improved water stability and resilience properties with additives, further research is necessary to enhance the foams’ hydrophobicity and resilience. Overall, the biobased foams, especially those with 1% FeCl_3_ and 1% CPAM, met and exceeded the minimum thermomechanical requirements of petroleum-based Type XI EPS insulation products, demonstrating their potential for building insulation and packaging applications.

## Figures and Tables

**Table 1:** Additives type and content used in the fabrication process of cellulose nanofibril-reinforced thermomechanical pulp fiber-based foams and their respective foam densities.

TMP Content (%)	CNF Content (%)	Additive Type	Additive Content * (pph)	Density (kg/m^3^)
		Neat low temp	0	34 (1%)
		Neat high temp	0	37 (5%)
95	5	Acrodur ^®^	1	36 (1%)
		Iron chloride	0.5	53 (4%)
		Iron chloride	1	70 (7%)
		CPAM	0.5	63 (3%)
		CPAM	1	94 (3%)

* Additives content was based on the total dry mass of the fibers. Values in parenthesis are coefficients of variation (CV%).

**Table 2:** Density and thermal conductivity of other foams.

Foam Type	Density (kg/m^3^)	Thermal Conductivity (W/m·K)	Reference
CNFs/TMP in x–y direction	100	0.046	[41]
CNFs/TMP in z direction	100	0.056	[41]
Pulp/Na_2_B_4_O_7_/CNFs	12	0.049	[12]
CNFs/starch	82	0.042	[53]
Pulp/chitosan/CPAM	53	0.068	[13]
Polyurethane modified with CNFs	50	0.044	[54]
Polyurethane	30–80	0.020–0.027	[55]
EPS	18–50	0.029–0.041	[55]

## Data Availability

Data are contained within the article.

## Supplementary Materials

The following supporting information can be downloaded at: https://www.mdpi.com/article/10.3390/nano14221837/s1, Figure S1: Scanning electron microscopy (SEM) images of the cross-sections of the cellulose nanofibril-reinforced thermomechanical pulp fiber-based foams with (a) no additives and (b) 1% Acrodur^®^, (c) 1% FeCl_3_, and (d) 1% CPAM as additives; Figure S2: Energy-dispersive X-ray spectroscopy (EDS) images of the cross-sections of the cellulose nanofibrils-reinforced thermomechanical pulp fiber-based foams with (a) 0.5% FeCl_3_, (b) 1% FeCl_3_, (c) 0.5% CPAM, and (d) 0.5% CPAM as additives: Figure S3: Fourier transform infrared (FTIR) spectra of the cellulose nanofibrils-reinforced thermomechanical pulp fiber-based foams of different formulations with and without different additives; Figure S4: The relationship between the compressive modulus and the density of the cellulose nanofibrils-reinforced thermomechanical pulp fiber-based foams of different formulations with and without different additives; Figure S5: The specific compressive modulus of the cellulose nanofibrils-reinforced thermomechanical pulp fiber-based foams of different formulations with and without different additives; Figure S6: Water stability of cellulose nanofibril films dried at (a) L: low temperature (12 h at 70 °C) and (b) H: high temperature (12 h at 70 °C+ 6 h at 105 °C) showed after stirring in DI water at 350 RPM for 20 and 50 min, respectively.
