# Supplementary material for: Enhancement of the Physical and Mechanical Properties of Cellulose Nanofibril-Reinforced Lignocellulosic Foams for Packaging and Building Applications
Source: Nanomaterials (Basel). 2024 Nov 17;14(22):1837. doi: 10.3390/nano14221837 (PMC11597808; doi:10.3390/nano14221837)
Supplement: Supplementary file 1 [file nanomaterials-14-01837-s001.zip › nanomaterials-3322703-supplementary.pdf]

Supplementary Information

Enhancement of the Physical and Mechanical Properties of Cellulose Nanofibril-Reinforced Lignocellulosic Foams for Packaging and Building Applications

Mara Paulette Alonso [1], Rakibul Hossain [2], Maryam El Hajam [2] and Mehdi Tajvidi [2,*]

[1] Department of Chemical and Biomolecular Engineering, University of California, Berkeley, CA 94720, USA; mpalonso@ncsu.edu
[2] School of Forest Resources and Advanced Structures and Composites Center, University of Maine, Orono, ME 04469, USA; rakibul.hossain@maine.edu (R.H.); maryam.el1@maine.edu (M.E.H.)
* Correspondence: mehdi.tajvidi@maine.edu

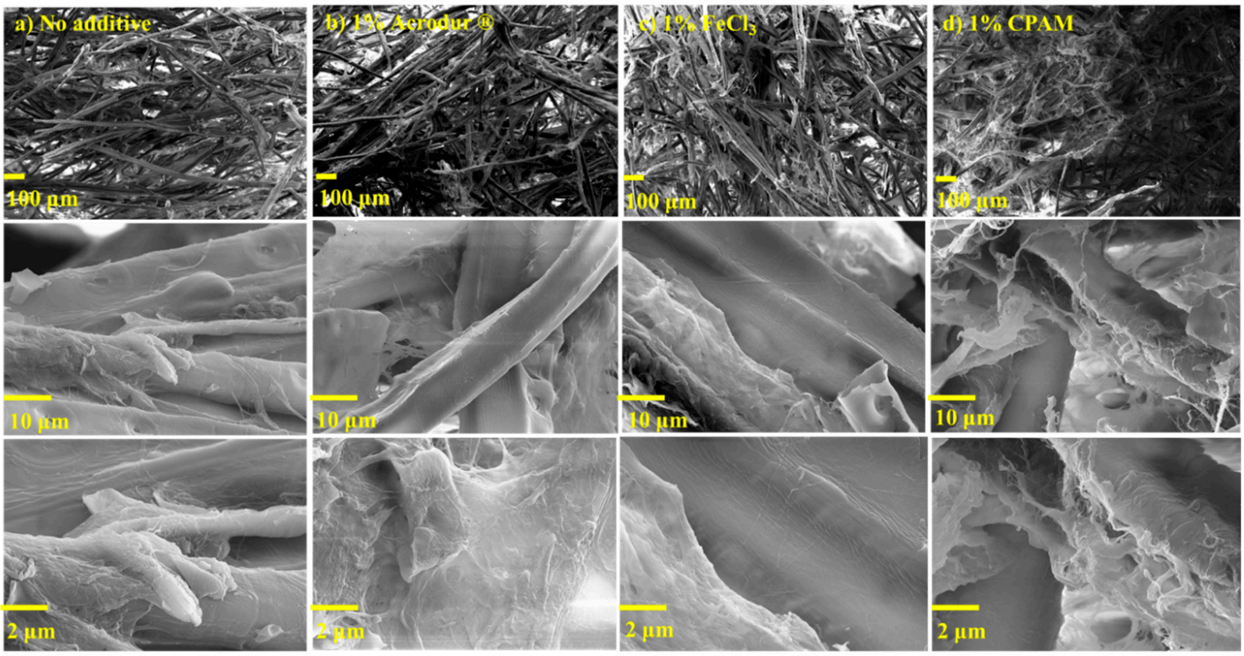

Figure S1. Scanning electron microscopy (SEM) images of the cross-sections of the cellulose nanofibril-reinforced thermomechanical pulp fiber-based foams with (**a**) no additives and (**b**) 1% Acrodur®, (**c**) 1% $FeCl_3$, and (**d**) 1% CPAM as additives.

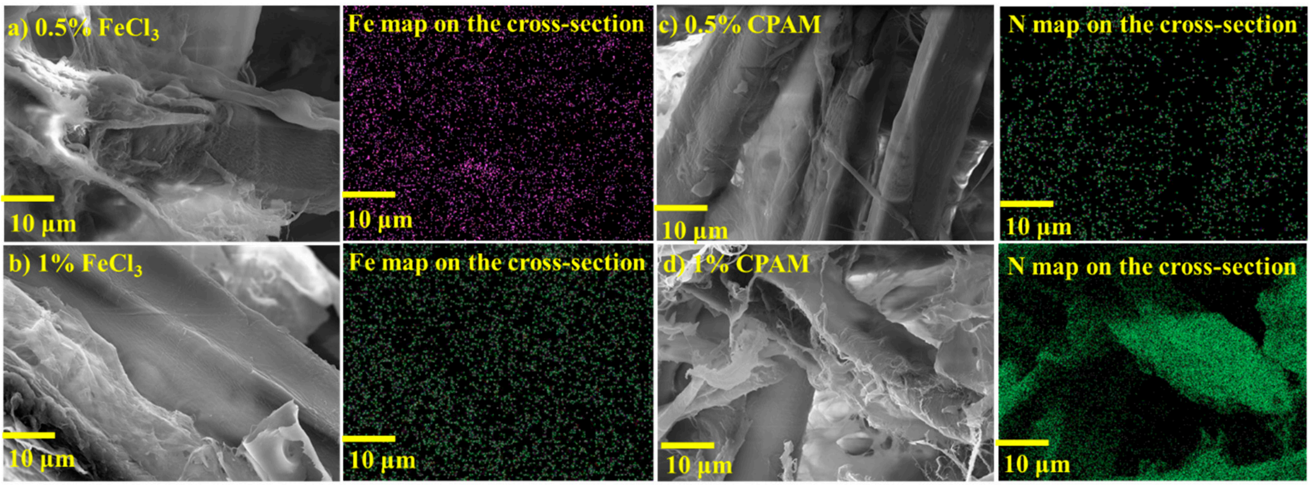

Figure S2. Energy-Dispersive X-ray Spectroscopy (EDS) images of the cross-sections of the cellulose nanofibrils-reinforced thermomechanical pulp fiber-based foams with (**a**) 0.5% $FeCl_3$ (**b**) 1% $FeCl_3$, (**c**) 0.5% CPAM, and (**d**) 1% CPAM as additives.

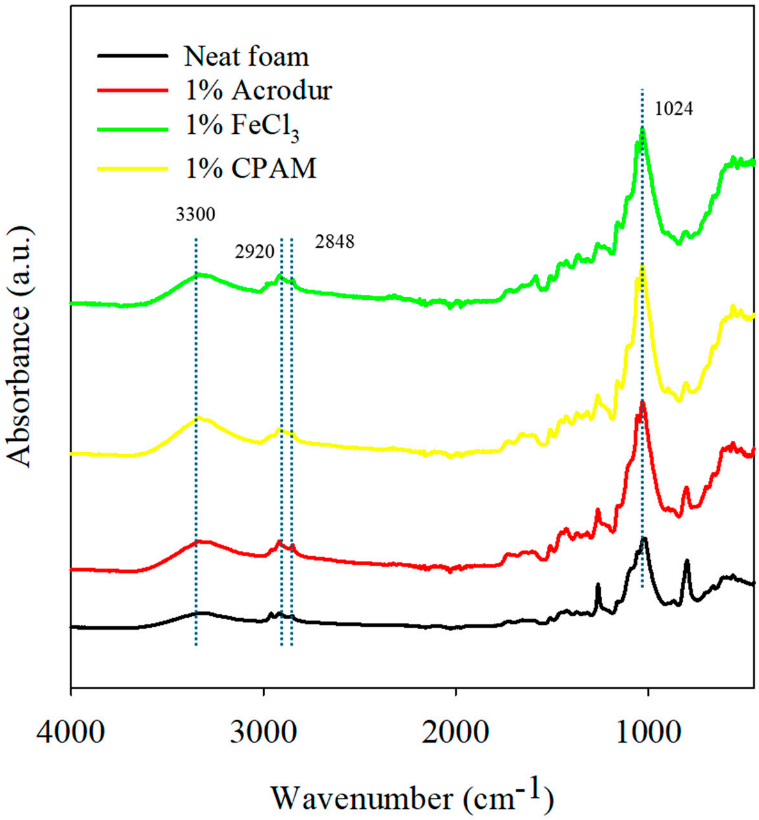

Figure S3. Fourier transform infrared (FTIR) spectra of the cellulose nanofibril-reinforced thermo-mechanical pulp fiber-based foams of different formulations with and without different additives.

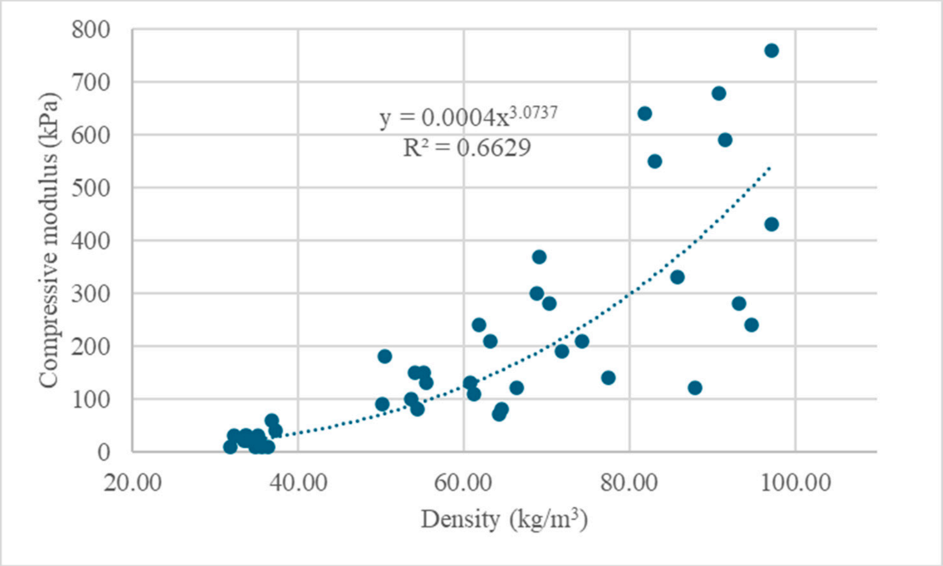

Figure S4. The relationship between the compressive modulus and the density of the cellulose nanofibrils-reinforced thermomechanical pulp fiber-based foams of different formulations with and without different additives.

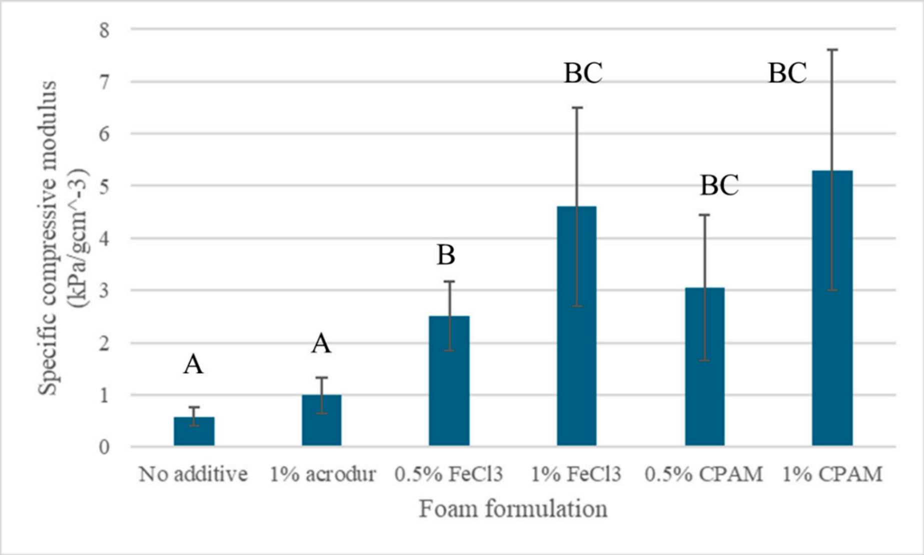

Figure S5. The specific compressive modulus of the cellulose nanofibrils-reinforced thermomechanical pulp fiber-based foams of different formulations with and without different additives.

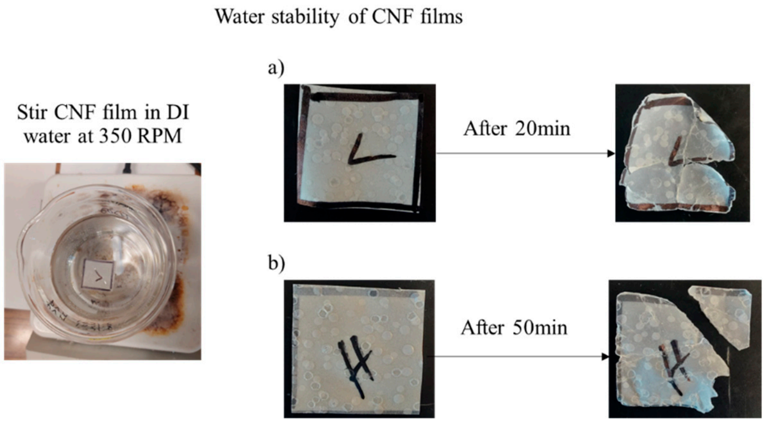

Figure S6. Water stability of cellulose nanofibril films dried at (**a**) L: low temperature (12h at 70°C) and (**b**) H: high temperature (12h at 70°C + 6h at 105°C) showed after stirring in DI water at 350 RPM for 20 and 50 minutes respectively.
